# Social Creativity and Entrepreneurial Intentions of College Students: Mediated by Career Adaptability and Moderated by Parental Entrepreneurial Background

**DOI:** 10.3389/fpsyg.2022.893351

**Published:** 2022-06-28

**Authors:** Libing Zhang, Qianqian Li, Ting Zhou, Chun Li, Chuanhua Gu, Xiuli Zhao

**Affiliations:** ^1^Key Laboratory of Adolescent Cyberpsychology and Behavior (CCNU), Ministry of Education, School of Psychology, Central China Normal University, Wuhan, China; ^2^Faculty of Education, Henan Normal University, Xinxiang, China; ^3^School of Education and Sports Science, Yangtze University, Jingzhou, China; ^4^Academy of Art and Design, Guangdong AIB Polytechnic, Guangzhou, China

**Keywords:** social creativity, entrepreneurial intention, career adaptability, college students, parental entrepreneurial background

## Abstract

Drawing on social cognitive career theory, this study aims to ascertain how social creativity influences college students’ entrepreneurial intentions, based on the mediating role of career adaptability and the moderating role of parental entrepreneurial background. A total of 715 college students completed an online survey designed to collect information on these variables. SPSS (version 25.0) was used to test the model. The results indicate that after controlling for gender and individual entrepreneurial experience, college students with a high level of social creativity were likely to have a high level of entrepreneurial intention. Career adaptability partially mediates the association between social creativity and entrepreneurial intention. Moreover, both direct and indirect associations between social creativity and entrepreneurial intention were moderated by parental entrepreneurial background. Specifically, compared with college students whose parents had no entrepreneurial background, the relationships between social creativity and entrepreneurial intention, social creativity and career adaptability, and career adaptability and entrepreneurial intention were stronger among college students whose parents had an entrepreneurial background. The findings help to develop promotion programs that are more suitable for college students’ entrepreneurship intentions.

## Introduction

Entrepreneurship can relieve employment pressure and promote economic development (Huang et al., 2021). College students are the vibrant force of high-level entrepreneurship owing to their high level of knowledge and innovative ability (Hu et al., 2018). Many countries have realized the importance of college students’ entrepreneurship in the context of the severe employment situation caused by the COVID-19 pandemic (Zhao et al., 2020). Recently, the State Council of China issued a policy to support college students’ entrepreneurship (The General Office of the State Council, 2021). Entrepreneurship is a process that goes from idea to practice, and from intention to implementation (Kong et al., 2020). Entrepreneurial intention can predict entrepreneurial behavior after 20 years (Schröder et al., 2011), which refers to an individual’s willingness to engage in entrepreneurial activities and strive to succeed entrepreneurially (Liñán and Chen, 2009). Given that entrepreneurial intention is regarded as the first step in entrepreneurship (Krueger et al., 2000) and the best predictor of entrepreneurial behavior (Ajzen, 1991), it is particularly valuable to examine the entrepreneurial intentions of college students to improve their entrepreneurial behaviors.

Among the numerous influencing factors of entrepreneurial intentions, creativity has attracted researchers’ attention (Zampetakis and Moustakis, 2006; Hamidi et al., 2008; Zampetakis et al., 2011; Chia and Liang, 2016). Creativity is the ability to produce products or create novel and useful ideas (Runco, 2004; Runco and Jaeger, 2012). Individuals with higher creativity have more innovative ideas and are more likely to start a business (Gorman et al., 1997). Researchers have suggested that creativity includes both domain generality (basic conditions required in all creative work) and domain specificity (based on the basic conditions, the conditions required for creative work in a certain field, such as artistic and social fields) (Baer and Kaufman, 2005; Sternberg, 2005; Ji and Gu, 2011). As creativity in the social field, social creativity aims to creatively solve problems in daily interactions and social activities (Mouchiroud and Bernoussi, 2008; Gu et al., 2016). Although empirical studies have verified the direct relationship between creativity and entrepreneurial intention (Zampetakis and Moustakis, 2006; Hamidi et al., 2008; Zampetakis et al., 2011; Chia and Liang, 2016), the research on the relationship between social creativity and entrepreneurial intention is still scant. From a broad perspective, entrepreneurship is a business activity that creates economic profits through human interaction in the social field (Mei et al., 2016), which may also be closely related to social creativity. Therefore, examining the relationship between social creativity and entrepreneurial intention may help improve college students’ entrepreneurial intentions.

Career adaptability (McKenna et al., 2016; Liang and Yi, 2017) and parental entrepreneurial background (Lindquist et al., 2015) have also been identified as antecedent variables of entrepreneurial intention. Empirical research shows that creativity is positively correlated with career adaptability (Eun, 2019). Additionally, previous studies on entrepreneurial intentions have reported the mediating role of career adaptability (Su and Qiao, 2019) and the moderating role of parental entrepreneurial background (Tolentino et al., 2014). Because few studies have examined the relationship between social creativity and entrepreneurial intention, the mediation mechanism behind the relationship (i.e., how does social creativity relate to college students’ entrepreneurial intention?) and the moderating mechanism (i.e., when is this connection stronger?) have not yet been discussed. The answers to these questions are related to our understanding of the influencing factors of and development promotion programs that are more suitable for college students’ entrepreneurship intentions. Considering research gaps and practical needs, career adaptability (mediator) and parental entrepreneurial intention (moderator) were introduced to examine the following three issues. First, whether college students with high social creativity have strong entrepreneurial intention. Second, whether social creativity can promote college students’ entrepreneurial intentions by increasing their career adaptability. Third, whether the relationship between social creativity and college students’ entrepreneurial intentions will vary owing to differences in parental entrepreneurial backgrounds.

## Literature Review and Hypothesis Development

### Social Creativity and Entrepreneurial Intention

Innovation theory indicates that entrepreneurship is essentially a form of “creative destruction,” and that creativity is the key factor (Schumpeter, 1934). Creativity is the ability to produce products or create novel and useful ideas (Runco, 2004; Runco and Jaeger, 2012). Novelty and usefulness are the core components of creativity and are the two main evaluation criteria generally accepted by researchers (Hennessey and Amabile, 2010; Bott et al., 2014; Abraham, 2018). Novelty refers to the degree to which an idea is rare and unusual (Dean et al., 2006), and only a few people can think of it (Runco and Charles, 1993; Diedrich et al., 2015). Novelty is a key feature that distinguishes creativity from ideas that are only considered good (Amabile et al., 2005). Usefulness reflects the feasibility, applicability, and rationality of an idea (Long, 2014). Creative work needs to meet both of the above evaluation dimensions; in other words, to be considered creative, an idea must be novel and simultaneously feasible rather than just unusual or novel (Rietzschel, 2005). Entrepreneurship is the process of building a new business (Gartner, 1985), which is novel and useful (Zampetakis and Moustakis, 2006). Therefore, entrepreneurship is relatively the result of creativity (Sternberg and Lubart, 1999). Many empirical studies have shown that creativity is significantly and positively correlated with entrepreneurial intention (Zampetakis and Moustakis, 2006; Hamidi et al., 2008; Zampetakis et al., 2011; Chia and Liang, 2016; Hu et al., 2018; Kumar and Shukla, 2019; Shi et al., 2020).

Researchers have suggested that creativity includes both domain generality and specificity (Sternberg, 2005; Ji and Gu, 2011). Baer and Kaufman (2005) proposed an amusement park theoretical (APT) model of creativity, which bridged the generality and specificity of creativity. According to the amusement park theoretical model of creativity, the basic requirement in all creative work is general creativity, and the specific requirement for creative work in a certain field, based on the basic conditions, is special creativity (i.e., artistic creativity and social creativity) (Baer and Kaufman, 2005). As a kind of creativity in the social field, social creativity is the quality of solving problems in the social field in original, appropriate, and effective ways (Mouchiroud and Bernoussi, 2008; Gu et al., 2016).

Compared with general creativity, social creativity not only has the basic characteristics of novelty and usefulness that contribute to entrepreneurial intentions but also has its particularity. First, social creativity aims to solve problems in people’s daily social activities. Moreover, social problem situations cover almost all people’s conscious social activities (Gu et al., 2015). Although economic profit maximization is the ultimate goal of entrepreneurship, from a broad perspective, entrepreneurship is a form of business activity that generates economic profits through human interaction in the social field (Mei et al., 2016). Entrepreneurship may also be closely related to social creativity. Second, individuals with high social creativity have strong dominance and autonomy, which helps improve their ability to lead and organize. These are necessary qualities for entrepreneurs that have been confirmed to promote entrepreneurial intentions (Mouchiroud and Lubart, 2002; Li and Li, 2017). Based on the aforementioned, although there is no research to test the relationship between social creativity and entrepreneurial intention directly, we can reasonably speculate that social creativity is positively correlated with college students’ entrepreneurial intentions (Hypothesis 1).

### The Mediating Role of Career Adaptability

College is an important transition stage from school to working. The development of college students’ career adaptability affects their career development throughout their lives (Wu et al., 2016). As the central concept of career construction theory, career adaptability refers to an individual’s positive psychological resources in managing current and anticipated vocational tasks, occupational transitions, and work trauma (Savickas and Porfeli, 2012). According to career construction theory, as a positive psychological resource, career adaptability is dynamic and highly malleable (Tolentino et al., 2014). Moreover, it is affected not only by individual characteristics but also by individuals’ career development (Guan and Li, 2015). Previous empirical studies used career adaptability as a mediating variable to explore the association between proactive personality and entrepreneurial intention (Zhang and Chen, 2021). Career adaptability mediates the relationship between Machiavellianism and entrepreneurial intention (Su and Qiao, 2019). Zhang et al. (2018) found that career adaptability mediates the effects of ambiguity tolerance on career decision-making difficulties. Career adaptability also plays a mediating role between competence flexibility and career decision-making difficulties (Leung et al., 2021). In summary, career adaptability plays a critical mediating role in existing empirical research.

Additionally, regarding the relationship between career adaptability and entrepreneurial intention, Savickas (2013) proposed that career adaptability can help individuals better cope with uncertainties and changes in the career environment. Regarding the uncertain career development path of entrepreneurship, the aforementioned advantages of career adaptability may contribute to an individual’s entrepreneurial intention. Empirical studies show that career adaptability is positively correlated with entrepreneurial intention (McKenna et al., 2016). Liang and Yi (2017) pointed out that career adaptability is a significant predictor of entrepreneurial intention. Individuals with strong career adaptability have stronger entrepreneurial intentions (Liang and Wang, 2016).

Regarding the relationship between social creativity and career adaptability, although no research has directly examined the relationship between the two variables, the relationship is indirectly supported by evidence. First, creativity is positively correlated with career adaptability (Eun, 2019), and social creativity has the basic characteristics of creativity (Gu et al., 2016). Therefore, social creativity may be positively correlated with career adaptability. Second, individuals with strong social creativity show the characteristics of initiative and risk-taking and can take decisive actions to adapt to and influence their career environment, which is positively correlated with career adaptability (Gu et al., 2008a; Zhang and Chen, 2021), again showing that social creativity may be positively related to career adaptability. Based on the above analysis, we can reasonably speculate that career adaptability mediates the relationship between social creativity and entrepreneurial intentions (Hypothesis 2).

### The Moderating Role of Parental Entrepreneurial Background

While social creativity can directly and/or indirectly influence college students’ entrepreneurial intentions through career adaptability, it does not influence all college students equally. Social cognitive career theory proposes that intention is the result of interaction between individuals and their environment (Lent et al., 1994). According to this theory, college students’ entrepreneurial intentions are affected by both individual and environmental factors. Existing research shows that the direct and indirect relationships between creativity and entrepreneurial intention vary in different entrepreneurial environments. When individuals are in a good entrepreneurial environment, the association between creativity and entrepreneurial intentions is stronger. Moreover, the interaction between the entrepreneurial environment and individual factors affects the strength of entrepreneurial intentions through mediating variables (Shen et al., 2018). Therefore, it is particularly important to examine the moderating effects of environmental factors on social creativity and individual entrepreneurial intentions.

This study confirms the moderating role of parental entrepreneurial background for several reasons. First, parental entrepreneurial background is an important environmental factor for college students. Parents’ professional role models affect their decisions to start a business (Schröder et al., 2011; Bosma et al., 2012). Individuals’ entrepreneurial intentions increase by approximately 60% if their parents have an entrepreneurial background (Lindquist et al., 2015). The influence of parents’ professional role models is most powerful at college (Bloemen-Bekx et al., 2019). More importantly, Chinese college students’ entrepreneurship intentions are easily affected by their family environment (especially parents) (Cao et al., 2020). Second, previous studies on entrepreneurial intention show that the moderating role of variables related to parental entrepreneurial backgrounds (Baluku et al., 2020; Brownhilder, 2020). For instance, a family entrepreneurial background plays a moderating role between entrepreneurship education and entrepreneurial intention (Bouhalleb, 2020). Family entrepreneurial traditions moderate the association between entrepreneurial self-efficacy and entrepreneurial intention (Mei et al., 2016).

Parental entrepreneurial background means that one or both parents of an individual have entrepreneurial experience or are in the process of starting a business (Palmer et al., 2019). Parents with entrepreneurial backgrounds can not only provide learning objects for their children, but also consciously encourage them to start a business and provide experience opportunities, guidance, and support in entrepreneurship (Lent et al., 1994; Bosma et al., 2012). The direct or indirect relationship between social creativity and entrepreneurial intention may vary according to differences in parental entrepreneurial backgrounds.

First, the direct relationship between social creativity and entrepreneurial intention differs in different contexts. A previous study found that when individuals perceive a better entrepreneurial environment, their creativity has a greater positive predictive effect on their entrepreneurial intention (Shen et al., 2018). In other words, individuals with high creativity have strong entrepreneurial intentions owing to the various forms of support provided by their entrepreneurial environment. Based on the results of the empirical research, we can reasonably speculate that parental entrepreneurial background moderates the relationship between social creativity and college students’ entrepreneurial intentions (Hypothesis 3a).

Second, the relationship between career adaptability and entrepreneurial intention differs in different entrepreneurial contexts. As a positive predictor of entrepreneurial intention (McKenna et al., 2016; Liang and Yi, 2017; Su and Qiao, 2019), individuals with strong career adaptability have higher entrepreneurial intentions if they have an entrepreneurial family background (Tolentino et al., 2014). In other words, a family entrepreneurial background enhances the entrepreneurial intentions of individuals with high career adaptability. Drawing on the findings of previous studies, we can reasonably speculate that parental entrepreneurial background moderates the relationship between career adaptability and college students’ entrepreneurial intentions (Hypothesis 3b).

Third, the relationship between social creativity and career adaptability differs across entrepreneurial contexts. According to social cognitive career theory and empirical research on social creativity, social creativity (individual factors) and parental entrepreneurial background (environmental factors) may jointly influence entrepreneurial intention (Lent et al., 1994; Gu et al., 2012). In other words, if college students with high creativity are supported by parents with entrepreneurial backgrounds, their career adaptability may also be strong. As such, we speculate that parental entrepreneurial background moderates the relationship between social creativity and the career adaptability of college students (Hypothesis 3c).

### Conceptual Model

**FIGURE 1:**
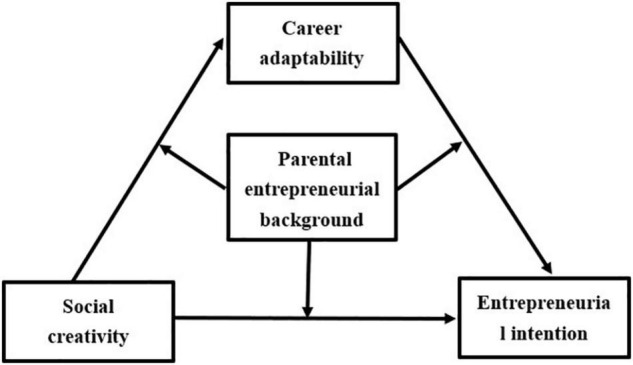
Theoretical hypothesis model.

Drawing on social cognitive career theory, this study constructs a moderated mediation model (Figure 1) to discuss the relationship between social creativity and college students’ entrepreneurial intentions and to explain how social creativity relates to the entrepreneurial intentions of college students and when the relationship is stronger. The specific hypotheses of the theoretical model are as follows:

Hypothesis 1: Social creativity is positively correlated with entrepreneurial intention.

Hypothesis 2: Career adaptability positively mediate the relationship between social creativity and entrepreneurial intention.

Hypothesis 3a: Parental entrepreneurial background positively moderate the relationship between social creativity and college students’ entrepreneurial intentions.

Hypothesis 3b: Parental entrepreneurial background positively moderate the relationship between career adaptability and college students’ entrepreneurial intentions.

Hypothesis 3c: Parental entrepreneurial background positively moderate the relationship between social creativity and college students’ career adaptability.

## Materials and Methods

### Participants

We recruited 800 participants from four universities in China using random cluster sampling. All the participants completed online questionnaires, and 715 valid questionnaires were retained, with an effective questionnaire recovery rate of 89%. Female students accounted for 52% (*n* = 372) of the sample. Parents of 35.8% of the participants (*n* = 256) had an entrepreneurial background. Additionally, 11.30% (*n* = 81) and 88.70% (*n* = 634) of the participants had or had no entrepreneurial experience, respectively.

### Measurements

#### Social Creativity

Social creativity was evaluated using Hu’s (2008) College Student Social Creativity Questionnaire. This questionnaire included 27 items assessing six dimensions of social creativity (e.g., “When solving problems in interpersonal communication, I can think of solutions from multiple angles and be flexible”). Each item is rated on a five-point scale (1 = strongly disagree, 5 = strongly agree). This measurement has demonstrated good reliability and validity among Chinese college students (Gu et al., 2015). In this study, the α coefficient for this questionnaire was 0.90. The fit index of the confirmatory factor analysis of the scale (χ^2^/df = 2.93, SRMR = 0.05, RMSEA = 0.05, CFI/TLI = 0.92) showed that the construct validity of the questionnaire was acceptable.

#### Career Adaptability

Career adaptability was evaluated using the Chinese version of the Career Adaptability Scale revised by Hou et al. (2012). This scale includes 24 items assessing four dimensions of career adaptability (e.g., “I have a plan on how to achieve my career goals”), and each item is rated on a five-point scale (1 = strongly disagree, 5 = strongly agree). This scale has been widely used to evaluate career adaptability and has good reliability and validity (Zhang et al., 2018; Su and Qiao, 2019). In this study, the α coefficient for this scale was 0.91. The fit index of the confirmatory factor analysis of the scale (χ^2^/df = 3.49, SRMR = 0.05, RMSEA = 0.06, CFI/TLI = 0.91) showed that the construct validity of the scale was acceptable.

#### Entrepreneurial Intention

Entrepreneurial intention was evaluated using an entrepreneurial intention scale developed by Liñán and Chen (2009). This scale includes six items assessing the single dimension of entrepreneurial intention (e.g., “My career goal is to become an entrepreneur”), and each item is rated using a five-point scale (1 = strongly disagree, 5 = strongly agree). This scale has been widely used to evaluate the entrepreneurial intention of individuals at home and abroad and has good reliability and validity (Shahab et al., 2018; Zhang and Zhang, 2018). In this study, the α coefficient for this scale was 0.91. The fit index of the confirmatory factor analysis of the scale (χ^2^/df = 2.44, SRMR = 0.01, RMSEA = 0.05, CFI/TLI = 0.99) showed that the construct validity of the scale was acceptable.

#### Parental Entrepreneurial Background

Parental entrepreneurial background was dichotomous and dummy coded. Participants whose parents had no entrepreneurial background were coded as 0. Participants whose parents had an entrepreneurial background were coded as 1.

#### Control Variables

According to existing research, an individual’s gender and entrepreneurial experience are related to their entrepreneurial intention (Zhao et al., 2005; Uddin et al., 2016; Bloemen-Bekx et al., 2019; Bignotti and le Roux, 2020). Therefore, we controlled for these variables in the statistical analyses to test the unique role of social creativity. Gender and entrepreneurial experience were dichotomous variables and dummy coded (0 = male; 1 = female; 0 = without entrepreneurial experience; 1 = with entrepreneurial experience).

### Procedure

Our institution’s Research Ethics Committee approved all the procedures. Participants voluntarily completed online questionnaires in the survey of the study without payment. This study used SPSS 25.0 and SPSS PROCESS macro (Hayes, 2013) to analyze the data and test the model. The variables used in the model were standardized before the analysis, and the control variables were considered in all the analyses.

## Results

### Common Method Bias

As the method of collecting data by self-reporting may result in common method deviations, this study conducts anonymous surveys and reverse scoring of some items to relatively control it. Before conducting the data analyses, we applied Harman’s single-factor test to check for common method bias. The results showed that a single factor explained 21.61% of the total variance, which was significantly lower than the critical standard of 40% (Zhou and Long, 2004). This indicated that there was no serious common method bias in this study.

### Preliminary Analyses

Table 1 shows the descriptive statistics and Pearson product-moment correlation coefficients for the study variables. Specifically, social creativity (*r* = 0.46, *p* < 0.001) and career adaptability (*r* = 0.48, *p* < 0.001) were significantly and positively associated with entrepreneurial intention, while social creativity was significantly and positively associated with career adaptability (*r* = 0.32, *p* < 0.001).

**TABLE 1:** Descriptive statistics and correlations for variables.

Variables	*M*	SD	1	2	3	4
1. Social creativity	3.12	0.43	1			
2. Career adaptability	2.87	0.43	0.32***	1		
3. Entrepreneurial intention	2.01	0.61	0.46***	0.48***	1	
4. Parental entrepreneurial background	–	–	0.30**	0.21**	0.33**	1

N = 715, ***p < 0.001, **p < 0.01.

### Mediation Analysis

To control for gender and individual entrepreneurial experience, we used PROCESS macro (Model 4) to test the mediation model with career adaptability as the mediating variable. Table 2 presents the results of the mediation analysis. Social creativity significantly positively predicted entrepreneurial intention (β = 0.46, *t* = 13.83, *p* < 0.001, 95% CI = [0.39, 0.53]). Career adaptability significantly and positively predicted entrepreneurial intention (β = 0.37, *t* = 11.48, *p* < 0.001, 95% CI = [0.31, 0.43]). Career adaptability significantly and positively predicted social creativity (β = 0.32, *t* = 8.92, *p* < 0.001, 95% CI = [0.25, 0.39]). Therefore, Hypothesis 1 is supported. Social creativity still significantly positively predicted entrepreneurial intention when the mediating variable of career adaptability was included (β = 0.34, *t* = 10.63, *p* < 0.001, 95% CI = [0.28, 0.41]). The results indicate that the link between social creativity and entrepreneurial intention was partially mediated by career adaptability (indirect effect = 0.12, 95% CI = [0.08, 0.16]). The mediation effect accounted for 26.09% of the total effect of social creativity on entrepreneurial intention. Therefore, Hypothesis 2 is supported.

**TABLE 2:** Testing the mediation model.

Model		Fitting index			Significance of coefficient			
outcome	Predictors	*R*	*R* ^2^	*F*	β	*LLCI*	*ULCI*	*t*
Entrepreneurial intention		0.46	0.21	64.28				
	Gender				−0.02	−0.15	0.11	−0.35
	Entrepreneurial experience				0.13	−0.07	0.34	1.25
	Social creativity (SC)				0.46	0.39	0.53	13.83***
Career adaptability		0.32	0.10	26.92***				
	Gender				0.07	−0.07	0.21	0.94
	Entrepreneurial experience				−0.06	−0.28	0.16	−0.58
	Social creativity (SC)				0.32	0.25	0.39	8.92***
Entrepreneurial intention		0.58	0.34	90.05***				
	Gender				−0.05	−0.17	0.07	−0.78
	Entrepreneurial experience				0.16	−0.03	0.34	1.61
	Social creativity (SC)				0.34	0.28	0.41	10.63***
	Career adaptability (CA)				0.37	0.31	0.43	11.48***

N = 715, *p < 0.05, **p < 0.01, ***p < 0.001. LLCI, lower limit of the 95% confidence interval; ULCI, upper limit of the 95% confidence interval; Gender, Entrepreneurial experience and Parental entrepreneurial background were dichotomous variables and were dummy coded (0 = male; 1 = female; 0 = without entrepreneurial experience; 1 = with entrepreneurial experience; 0 = parents without entrepreneurial background; 1 = parents with entrepreneurial background). All the variables in the model were standardized.

### Moderation Analysis

To control for gender and individual entrepreneurial experience, we used the PROCESS macro (Model 59) to test the moderating effect of parental entrepreneurial backgrounds. Table 3 shows the results. Social creativity interacting with parental entrepreneurial background can significantly and positively predict entrepreneurial intention and career adaptability (β = 0.17, *p* < 0.05; β = 0.18, *p* < 0.05), and career adaptability interacting with parental entrepreneurial background can significantly and positively predict entrepreneurial intention (β = 0.15, *p* < 0.05). Both the direct and indirect relationships between social creativity and entrepreneurial intention were moderated by parental entrepreneurial backgrounds. Therefore, Hypotheses 3a, 3b, and 3c are supported.

**TABLE 3:** Testing the moderation model.

Model		Fitting index			Significance of coefficient			
outcome	Predictors	*R*	*R* ^2^	*F*	β	*LLCI*	*ULCI*	*t*
Career adaptability		0.35	0.12	20.04***				
	Gender				0.07	−0.07	0.21	1.01
	Entrepreneurial experience				−0.07	−0.29	0.15	−0.64
	Social creativity (SC)				0.22	0.13	0.31	4.70***
	Parental entrepreneurial background (PEB)				0.24	0.09	0.39	3.07***
	SC × PEB				0.18	0.02	0.33	2.28*
Entrepreneurial intention		0.61	0.38	60.78***				
	Gender				−0.03	−0.15	0.08	−0.54
	Entrepreneurial experience				0.15	−0.03	0.34	1.61
	Social creativity (SC)				0.24	0.16	0.32	5.97***
	Career adaptability (CA)				0.29	0.21	0.36	7.26***
	Parental entrepreneurial background (PEB)				0.55	0.67	0.43	6.34***
	SC × PEB				0.17	0.03	0.30	2.39*
	CA × PEB				0.15	0.02	0.29	2.32*

N = 715, *p < 0.05, ***p < 0.001. LLCI, lower limit of the 95% confidence interval; ULCI, upper limit of the 95% confidence interval; Gender, Entrepreneurial experience and Parental entrepreneurial background were dichotomous variables and were dummy coded (0 = male; 1 = female; 0 = without entrepreneurial experience; 1 = with entrepreneurial experience; 0 = parents without entrepreneurial background; 1 = parents with entrepreneurial background). All the variables in the model were standardized.

**FIGURE 2:**
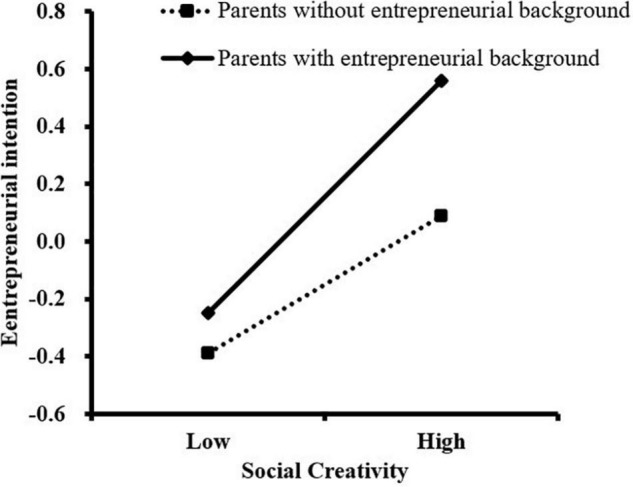
The moderating effect of parental entrepreneurial background on the relationship between social creativity and entrepreneurial intention.

**FIGURE 3:**
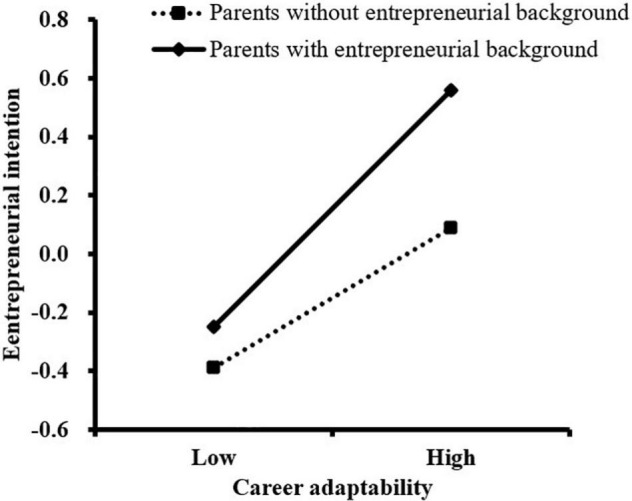
The moderating effect of parental entrepreneurial background on the relationship between career adaptability and entrepreneurial intention.

**FIGURE 4:**
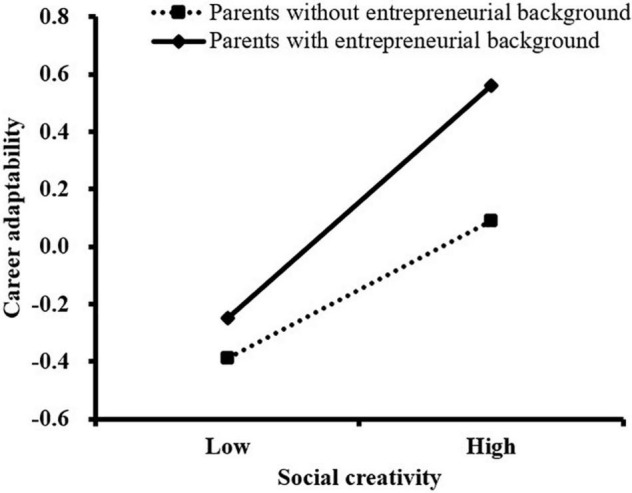
The moderating effect of parental entrepreneurial background on the relationship between social creativity and career adaptability.

We further conducted a simple slope analysis to analyze the moderating effect of parental entrepreneurial background in this study. As shown in Figure 2, for college students whose parents had an entrepreneurial background, the association between social creativity and entrepreneurial intention was stronger (β*_*simple*_* = 0.40, *t* = 7.15, *P* < 0.001) than for those whose parents did not have an entrepreneurial background (β*_*simple*_* = 0.24, *t* = 5.97, *P* < 0.001). As illustrated in Figures 3, 4, the association between career adaptability and entrepreneurial intention, as well as the association between social creativity and career adaptability, was more remarkable for college students whose parents had entrepreneurial background (β*_*simple*_* = 0.44, *t* = 8.18, *P* < 0.001; β*_*simple*_* = 0.22, *t* = 4.70, *P* < 0.001), compared with those whose parents had no entrepreneurial background (β*_*simple*_* = 0.29, *t* = 7.26, *P* < 0.001; β*_*simple*_* = 0.39, *t* = 6.34, *P* < 0.001).

In summary, career adaptability mediates the relationship between social creativity and entrepreneurial intention. Both direct and indirect associations between social creativity and entrepreneurial intention were moderated by parental entrepreneurial background. All the hypotheses of this study are supported (see Table 4).

**TABLE 4:** Results of hypothesis testing.

Hypothesis	Relationship		β	*t*	*p*	Decision
H1	SC→EI		0.46	13.83	0.001	Yes
		SC→CA	0.32	8.92	0.001	
H2	SC→CA→EI	CA→EI	0.37	11.48	0.001	Yes
		SC→EI	0.34	10.63	0.001	
H3a	SC × PEB→EI		0.17	2.39	0.05	Yes
H3b	CA × PEB→EI		0.15	2.32	0.05	Yes
H3c	SC × PEB→CA		0.18	2.28	0.05	Yes

*SC, social creativity; CA, career adaptability; EI, entrepreneurial intention; PEB, parental entrepreneurial background.*

## Discussion

Although many empirical studies have verified that creativity is significantly positively related to entrepreneurial intention (Zampetakis and Moustakis, 2006; Hamidi et al., 2008; Zampetakis et al., 2011; Chia and Liang, 2016; Hu et al., 2018; Kumar and Shukla, 2019; Shi et al., 2020), the relationship between social creativity and entrepreneurial intention remains significantly unclear. We draw on social cognitive career theory to confirm the relationship between social creativity and entrepreneurial intention, as well as the mediating and moderating mechanisms underlying this relationship, by proposing and testing a moderated mediation model. The results showed that social creativity was significantly and positively associated with college students’ entrepreneurial intentions and that this association was mediated by career adaptability. More importantly, both the direct and indirect relationships between social creativity and college students’ entrepreneurial intentions were moderated by their parental entrepreneurial background.

### Social Activity and Entrepreneurial Intention

As predicted in Hypothesis 1, social creativity is significantly and positively associated with college students’ entrepreneurial intentions. In other words, college students with higher social creativity have higher entrepreneurial intentions, which is consistent with the conclusions of existing research on general creativity (Hamidi et al., 2008; Zampetakis et al., 2011; Laguia et al., 2018; Kumar and Shukla, 2019; Shi et al., 2020). This result supports the view that creativity is important for entrepreneurship in Schumpeter’s innovation theory (Schumpeter, 1934). This phenomenon can be explained in two ways. On the one hand, individuals with high social creativity have more novel and useful ideas, are curious about new things, and are willing to take risks (Gu et al., 2016). These qualities are essential for entrepreneurs (Kusmintarti et al., 2017), as they help individuals identify entrepreneurial opportunities, obtain resources, and allocate them rationally to start a new business (Zhang and Zhang, 2018). On the other hand, the particularity of social creativity contributes to the generation of entrepreneurial intention. In the process of entrepreneurship, college students may encounter problems such as entrepreneurial financing, social support, and entrepreneurial guidance, which require good social skills and problem-solving capabilities (Han and Yang, 2020). College students with high social creativity are good at solving problems in these social fields (Mouchiroud and Bernoussi, 2008; Gu et al., 2016), which helps them generate entrepreneurial intentions. Therefore, college students with higher social creativity have stronger entrepreneurial intention.

This finding confirms the importance of social creativity in entrepreneurship. It is worth noting that previous studies using educational experiments have confirmed that social creativity can be cultivated and shaped through teaching (Gu, 2007). Additionally, social creativity is a form of creativity with strong popularity because it involves individuals’ daily social activities and many aspects of social life (Gu et al., 2015), prompting us to consider the cultivation and promotion of social creativity. From a personal point of view, enhancing social creativity can solve many specific problems in the entrepreneurial process and daily social activities of college students and help them succeed in their entrepreneurial careers. From the school’s point of view, incorporating the content of social creativity training to entrepreneurship education activities and courses will help increase the entrepreneurial intention of college students. Although this study is an initial exploration of the relationship between social creativity and entrepreneurial intention, its findings on their relationship provide empirical support for individual entrepreneurial development and entrepreneurship education in schools. Future research can also formulate specific teaching content for the cultivation of social creativity according to the problems involved in the field of entrepreneurship, and further test the application effect of social creativity in the field of entrepreneurship.

### The Mediating Role of Career Adaptability

The relationship between social creativity and the entrepreneurial intentions of college students is mediated by career adaptability, as predicted in Hypothesis 2. When college students have higher social creativity, they may have more positive psychological resources for their careers and stronger entrepreneurial intentions. This result indicates that, compared with social creativity, career adaptability is a proximal factor of entrepreneurial intention, which may be an important explanation mechanism for why individuals with high social creativity have stronger entrepreneurial intentions. This finding is congruent with previous studies showing that career adaptability mediates the relationship between personal traits and entrepreneurial intention (Su and Qiao, 2019; Zhang and Chen, 2021).

In addition to the overall mediation, each of the individual links in the mediation model is worth discussing. Regarding the relationship between social creativity and career adaptability, college students with higher social creativity have more novel and useful ideas, good social skills, and initiatives (Gu et al., 2008a,b). Individuals rationally construct the above-mentioned characteristics of social creativity and form internal positive psychological resources by adjusting themselves (Savickas, 2013). Therefore, individuals with higher social creativity have richer positive psychological resources, namely stronger career adaptability.

Moreover, in terms of the relationship between career adaptability and entrepreneurial intentions, career adaptability is a positive psychological resource, and individuals with this resource will accept and respond to uncertainty in entrepreneurship with an open mind, overcome the anxiety caused by ambiguity and unclearness in entrepreneurship, and transform from school to future workplace (entrepreneurship) smoothly, which can promote college students’ recognition of entrepreneurial activities and help the formation of entrepreneurial intention (Liang and Wang, 2016). Additionally, individuals with this resource exhibit career planning and exploration behaviors, strong tolerance for frustration, and the spirit of facing difficulties, which are necessary qualities for entrepreneurs, and help enhance entrepreneurial intentions (Guan and Li, 2015). Therefore, career adaptability bridges the relationship between social creativity and entrepreneurial intention.

Considering the important role of career adaptability in the field of entrepreneurship and its own moldability, it is necessary to incorporate career adaptability training into entrepreneurship education courses and help college students clarify and enhance their entrepreneurial intentions through systematic course teaching. A clear career planning goal is an important prerequisite for college students to choose entrepreneurship; however, the current entrepreneurship education courses in colleges and universities mainly focus on knowledge and skills and lack the relevant content of career planning (Zhang and Chen, 2021). Future entrepreneurship education courses can organically integrate the content of social creativity and career adaptability training, to further enhance the entrepreneurial intention of college students by reinforcing the cultivation of college students’ social creativity level, and conducting career adaptability training.

### The Moderating Role of Parental Entrepreneurial Background

This study shows that parental entrepreneurial background moderates both direct and indirect relationships between social creativity and college students’ entrepreneurial intentions. Hypotheses 3a, 3b, and 3c in this study are all supported. This result verifies the viewpoint of social cognitive career theory, which states that the interaction between environmental and individual factors influences individual career choices (Lent et al., 1994).

Parental entrepreneurial background, as an environmental factor, can influence college students’ entrepreneurial intentions together with individual factors because it can provide role model incentives, resource support, and financial support for college students (Bosma et al., 2012; Bae et al., 2014; Palmer et al., 2019). Specifically, regarding the relationship between social creativity and entrepreneurial intention, compared with college students whose parents have no entrepreneurial background, the relationship is stronger among college students whose parents have entrepreneurial background. This may be because college students with high social creativity can creatively solve problems in entrepreneurial activities. However, they still do not know new things about entrepreneurial activities well because they do not enter the workplace (Zhang and Chen, 2021). However, parents with entrepreneurial backgrounds are role models for individual learning, such as parents’ preferences for entrepreneurship and the interaction between entrepreneurs (Chlosta et al., 2012; Bae et al., 2014). They also appreciate their children’s autonomy in entrepreneurial careers, which stimulates college students’ entrepreneurial intentions (Bloemen-Bekx et al., 2019). The motivation of role models, based on their abilities, encourages college students to recognize their entrepreneurial identity and enhance their entrepreneurial intentions.

Regarding the relationship between social creativity and career adaptability, compared with college students whose parents have no entrepreneurial background, the relationship is stronger among college students whose parents have entrepreneurial backgrounds. One possible reason is that college students with high social creativity are open, initiative, and extroverts, which are conducive to social adaptation. Meanwhile, parents with entrepreneurial backgrounds can provide internship positions and experience guidance for entrepreneurship (such as entrepreneurial knowledge, skills, and opportunity identification) (Bosma et al., 2012). These experiences allow individuals to better face a new career environment. More importantly, college students who receive emotional support from parents with entrepreneurial backgrounds have better tolerance for entrepreneurial failures and setbacks, which is particularly important for improving career adaptability (Shirokova et al., 2015).

Regarding the relationship between career adaptability and entrepreneurial intention, compared with college students whose parents have no entrepreneurial background, the relationship is stronger among college students whose parents have entrepreneurial backgrounds. This may be because college students with high career adaptability can reasonably construct their entrepreneurial experience and abilities and form a positive attitude to cope with their future careers (Savickas, 2013). Moreover, many college students encounter financing difficulties (Han and Yang, 2020), one of the constraints to future entrepreneurship of college students, and parents with entrepreneurial backgrounds are more likely to provide corresponding financial support (Palmer et al., 2019). Therefore, college students with a positive attitude have stronger entrepreneurial intentions owing to the financial backup force from parents with an entrepreneurial background.

The moderating effect of parental entrepreneurial background confirms the boundary conditions of the relationship between social creativity and entrepreneurial intention. According to social career theory, in addition to teaching knowledge and skills, future school entrepreneurship education should fully consider the micro-environmental factors of individual entrepreneurship. Under the current situation of a large population and the era of mass entrepreneurship and innovation in China, entrepreneurship education in universities can consider grouping teaching according to different parental entrepreneurial backgrounds, provide personalized courses for the different entrepreneurial needs of college students, integrate resources to improve the efficiency and effect of entrepreneurship education, and alleviate the problem of broad entrepreneurship education and low entrepreneurship rates.

## Conclusion

This study aimed to investigate the influence of social creativity on college students’ entrepreneurial intentions with the mediating role of career adaptability and the moderating role of parental entrepreneurial background. The results show a critical mechanistic pathway (*via* career adaptability) through which higher social creativity was related to stronger entrepreneurial intention. Additionally, both direct and indirect associations between social creativity and entrepreneurial intention were moderated by parental entrepreneurial background. Compared with college students whose parents had no entrepreneurial background, the relationships between social creativity and entrepreneurial intention, social creativity and career adaptability, and career adaptability and entrepreneurial intention were stronger among college students whose parents had an entrepreneurial background. This study extends the research on the relationship between social creativity and college students’ entrepreneurial intentions.

## Implications

First, this study shows that social creativity significantly contributes to predicting college students’ entrepreneurial intentions, which enriches the literature on the influencing factors of entrepreneurial intentions and expands the scope of the application of social creativity. More importantly, it provides theoretical support for entrepreneurial intention promotion programs based on the cultivation of social creativity.

Second, career adaptability is a vital factor in connecting social creativity and entrepreneurial intention, which mediated the relationship between them. Previous research does not report an internal mechanism between social creativity and entrepreneurial intention, which undoubtedly limits the further expansion of college students’ entrepreneurial intention promotion programs. The mediating role of career adaptability confirmed in this study fills this gap. Furthermore, career adaptability is dynamic and malleable and can be improved through education and training (Savickas and Porfeli, 2012; Tolentino et al., 2014). Hence, it can be considered to enhance college students’ entrepreneurial intentions by improving their career adaptability.

Third, this research explores the relationship between social creativity and college students’ entrepreneurial intentions from the perspective of social cognitive career theory and career construction theory and confirms the key role of parental entrepreneurial background as an environmental factor. The results remind us that the improvement effect of college students’ entrepreneurial intention may be better if the characteristics of college students’ home environment, especially parental entrepreneurial background, are considered in the promotion programs of college students.

## Limitations and Future Directions

First, although the hypothesized conceptual models are based on relevant theories and empirical research, correlational study results may not conclusively prove the causal relationship between variables because the data were collected simultaneously. There is need for a longitudinal study to investigate college students’ entrepreneurial intentions at different stages and provide more abundant and accurate evidence for the relationship between social creativity and college students’ entrepreneurial intentions.

Second, the variables were collected using a questionnaire and subjective reports. Although there was no serious common method bias, the results were inevitably influenced by social expectations. Therefore, future studies should consider collecting data from multiple perspectives (such as peer nominations or teacher evaluations).

Third, this is the first study to investigate the moderating role of parental entrepreneurial background on the direct and indirect relationships between social creativity and entrepreneurial intention. The professional role models of parents may have different effects on their children’s future entrepreneurial intentions (Hoffmann et al., 2015). Therefore, future research should consider conducting a comparative study of college students’ entrepreneurial intentions based on their parents’ entrepreneurial backgrounds.

## Data Availability Statement

The raw data supporting the conclusions of this article will be made available by the authors, without undue reservation.

## Ethics Statement

All procedures performed in this study were approved by the Ethics in Human Research Committee of Central China Normal University. Written informed consent was obtained from all participants for their participation in this study.

## Author Contributions

LZ, TZ, and CG described and developed the review and hypothesis and formulated the study limitations and future directions for research. QL, CL, and XZ involved in the data collection process. LZ, QL, and XZ performed the analysis, interpretation of the results, and formulated the main conclusions. CG and XZ provided advices for the revision. XZ participated in specific text revisions. All authors helped to editing and formatting the manuscript, contributed to the article, and approved the submitted version.

## Conflict of Interest

The authors declare that the research was conducted in the absence of any commercial or financial relationships that could be construed as a potential conflict of interest.

## Publisher’s Note

All claims expressed in this article are solely those of the authors and do not necessarily represent those of their affiliated organizations, or those of the publisher, the editors and the reviewers. Any product that may be evaluated in this article, or claim that may be made by its manufacturer, is not guaranteed or endorsed by the publisher.
